# Association between water related factors and active trachoma in Hai district, Northern Tanzania

**DOI:** 10.1186/2049-9957-1-10

**Published:** 2012-11-01

**Authors:** Michael J Mahande, Humphrey D Mazigo, Eliningaya J Kweka

**Affiliations:** 1 Community Health Department, KCM College of Tumaini University, PO Box 2240, Moshi, Tanzania; 2 Department of Medical Parasitology and Entomology, Catholic University of Health and Allied Sciences, P.O. Box 1464, Mwanza, Tanzania; 3 Tropical Pesticides Research Institute, Division of Livestock and Human Disease vector control, PO Box 3024, Arusha, Tanzania

**Keywords:** Trachoma, Water, Hygiene, Tanzania

## Abstract

**Background:**

Trachoma is widely distributed in sub-Saharan Africa and is mainly associated with poor water accessibility. However, these associations have never been demonstrated in some of the communities, especially in northern Tanzania. To cover that gap, the present case control study was conducted to assess the association of water related factors, general hygiene and active trachoma among preschool and school age children in Hai district, northern Tanzania.

**Results:**

Families reported to use > 60 litres of water per day were less likely to have active disease (OR= 0.4, 95% CI: 0.1 - 0.3; *P*<0.001) compared to households collecting ≤ 60 litres. The risk of having trachoma increased with increase in distance to the water point (OR= 6.5, 95% CI; 1.8 - 16.7; *P*= 0.003). Households members who reported to use < 2 liters of water for face washing were more likely to be trachomatous (OR= 5.12, 95% CI: 1.87-14.6, *P* = 0.001). Increased number of preschool children in the household was also associated with increased risk of active trachoma by 2.46 folds.

**Conclusions:**

Improving water supply near the households and providing public health education focusing on improving households socio-economic status and individual hygiene especially in pre-school children in part will help to reduce the prevalence of the disease. In addition, integrating public health education with other interventions such as medical interventions remains important.

## Multilingual abstracts

Please see Additional file 1 for translations of the abstract into the six official working languages of the United Nations.

## Background

Globally, 1.2 billion people live in trachomatous endemic areas, in which, 40.6 million individuals have active trachoma and 8.2 million have trichiasis [1]. Trachoma is highly endemic in developing countries especially in areas with poor water supply [2]. In endemic countries, the disease is focally distributed and may only be confined to some communities or households within a single community [3]. A number of household’s factors have been previously described increase the transmission of the disease [3]. Factors such as poverty, poor water supply and availability and poor sanitation have been described as the risk factors for transmission of trachoma [3,4]. Similarly, the presence of flies, amount of water used for washing children, absence of latrine at a household and disposing of animal dung close to the households were identified as additional risk factors for trachoma transmission [5-11].

The associations between water sources and trachoma have remained to be a topic of debate [12]. Previous studies have reported a strong association between distances to water sources and trachoma among children [3,12,13]. In fact, In Mali, availability of water and use of water for hygiene practices such as washing face was reported to reduce the prevalence of active trachoma [10]. In contrast, other studies have reported a lack of association between a distance to the water source or presence of water source in a village and endemicity of trachoma [11,14,15].

World Health Organization and other partners jointly have called for the global elimination of blinding trachoma by 2020 (GET2020) [16]. The aim of the alliance is to eliminate the blindness caused by trachoma and the alliance agreed to promote SAFE (Surgery, Antibiotic therapy, Facial cleanliness and Environmental improvement) as the main community strategies to control trachoma [16]. In endemic areas, the main challenge to meet the GET2020 target is poverty. Poverty is associated with all the factors previously reported to be the main risk factors for trachoma transmission [6-11].

Importantly, the risk factors associated with trachoma transmission tends to vary from one epidemiological settings to another, thus to meet the GET2020 targets and plan for any public health interventions, it is important to understand the local specific risk factors associated with the disease transmission either at community or household levels. The key questions in the present study were to understand (i) if the amount of water collected differ between households with or without active trachoma? (ii) Is the distance (measured in term of time) to water sources differs between households with or without active trachoma? In addition, the study explored other households risk factors associated with active trachoma at Hai district, Northern Tanzania.

## Results

### Characteristics of the study participants

The size of sampled households ranged between 5–10 people with 2–5 children aged <10 years. Although the numbers of children were similar on case and control households, the number of pre-school children in case households was higher than in control households (χ2trend, **=** 5.8 P=0.02**)**.

### Sources of water for different use in the households

The main sources of water for the 96 respondents were river 39 (40.6%), public tap 26 (27.1%), stream/furrow 16(16.7%) and spring 15(15.6%). Collected water was mainly used for domestic use includes drinking, washing and cooking. In both households (with or without active trachoma), household mothers were the main decision makers on how the collected water was used in the household.

### Factors associated with trachoma at univariate analysis

Table 1 shows the risk factors and other household’s characteristics which were associated with trachoma among households with or without active trachoma. Factors such as increased frequency face washing among children (OR= 0.15; 95% CI: 0.05-0.42, *P*<0.001), the daily use of ≥ 2 litres of water for face washing (OR= 0.2, 95% CI, 0.07 – 0.71, *P*= 0.001), and bathing of children (OR= 0.05; 95% CI: 0.01-0.41, *P*= 0.001) were associated with reduced risk of active trachoma among control households. In fact, the households reported to collect ≥ 60 litres of water on daily basis were less likely to have trachoma (OR= 0.02, 95% CI, 0.01– 0.08, *P*= 0.001). For the households with active cases of trachoma, they reported a daily collection of 10–20 litres of water and reported low frequency of face washing and bathing of children compared to control households (Table 1). Interestingly, the distance to water source and amount of water collected in the family were independently associated with active trachoma. In additional, households reported to walk for 20 minutes to the water sources were nearly 6-fold at risk of having active trachoma. Furthermore, the presence of preschool children in the household was also associated with active trachoma (OR= 2.54, 95% CI: 1.24 - 6.12).

**Table 1:** Hygiene, sanitation and water-use factors associated with households with active disease’ (univariate analysis)

**Risk factors**	**Case household**	**Control household**		**OR**	**(95% CI)**	**P - value**
**Hygiene factors**						
Frequency of face washing			Total			
>= 2 a day	19 (39.6%)	39 (81.3%)	58 (60.4%)	0.15	(0.05-0.42)	<0.001
Once a day	29 (60.4%)	9 (18.8%)	38 (39.6%)	1		
Bathing of children						
Yes	34 (70.8%)	47 (97.9%)	81 (84.4%)	0.05	(0.01-0.41)	<0.001
No	14 29.12%)	1 (2.1%)	15 (15.6%)	1		
Decision making on water at home						
Mother	46 (95.8%)	47 (97.9%)	93 (96.7%)	0.49	0.02-7.21	NS
Father	2 (4.2%)	1 (2.1%)	3 (3.3%)	1		
Use of soap for hand washing						
Yes	34 (70.8%)	38 (77.0 %)	71 (74.0%)	0.72	0.29-1.81	NS
No	14 (29.2%)	11 (23.0%)	25 (26.0%)	1		
Use of soap for face washing						
Yes	36 (75.0%)	41 (85.0%)	77 (80.2%)	0.51	0.18-1.44	NS
No	12 (25.0%)	7 (15.0%)	19 (19.8%)	1		
Sharing of vessels during face washing						
Yes	13 (27%)	6 (12.5%)	19 19.8%)	2.6	0.89-7.55	NS
No	35 (73%)	42 (87.5%)	7 (80.2%)	1		
Sharing of towel						
Yes	9 (18.8%)	8 (16.7%)	17 (17.7%)	1.2	0.40-3.32	NS
No	39 (81.2%)	40 (83.3%)	79 (82.3%)	1		
**Water use in household**						
Daily household water for face washing						
>= 2 litres	22 (45.8%)	39 (81.2%)	61(63.5%)	0.2	0.07- 0.71	<0.001
1 Litre	26 (52.2%)	9 (18.8%)	35 (36.5%)	1		
Distance to water source (minutes)						
> 20 minutes	22 (45.8%)	6 (12.5%)	28 (29.2%)	5.92	1.93-18.96	<0.001
0-20 minutes	26 (52.2%)	42 (87.5%)	68 (70.8%)	1		
Household daily water collection (Litres)						
>=60	5 (10.4%)	41 (85.4%)	46 (47.9%)	0.02	0.01-0.08	<0.001
<60	43 (89.6%)	7 (14.6%)	50 (52.1%)	1		
Frequency of daily water collection (trips)						
>= 2 trips	38 (79.2%)	42 (87.5%)	80 (83.3%)	0.54	16-1.83	NS
1 trip	10 (20.8%)	6 (12.5%)	16 (16.7%)	1		
Daily household water for bathing (Litre)						
>20	13 (27.1%)	28 (58.3%)	41 (42.7%)	0.55	0.24-1.26	NS
10 - 20	35 (72.9%)	20 (41.7%)	55 (57.3%)	1		
Person who collect water						
Mother	37 (77.1%)	37 (77.1%)	74 (77.1%)	1.0	0.39-2.59	NS
Daughters	11 (22.9%)	11 (22.9%)	22 (22.9%)	1		
**Latrines and waste management**						
Presence of latrine at household						
Yes	30 (62.5%)	36 (75.0%)	66 (68.8%)	0.56	0.21-1.45	NS
No	18 (37.5%)	12 (25.0%)	30 (31.2%)	1		
Waste management system						
Present	20 (41.7%)	31 (64.6%)	51 (53.1%)	0.39	0.17-0.89	0.04
Absent	28 (58.3%)	17 (35.4%)	45 (46.9%)	1		
Number of pre-school children in household						
Yes	30 (61.2%)	19 (38.8%)	49 (51.0%)	2.54	1.24 - 6.12	0.03
no	18 (38.3%)	29 (61.7%)	47 (49.0%	1		

**Note**: A case household is a house with 2 or more children with active trachoma (TF).

**NS**: Non significant.

### Factors associated with active trachoma at multivariate analysis

On multivariate analysis, (Table 2) only the long distance to the water source and the presence of pre-school children in the households remained independently associated with active trachoma. However, other factors such as allocation of ≥ 2 litres of water daily for face washing showed protective effect against active trachoma. Other factors as shown in Table 2 were not associated with active trachoma.

**Table 2:** Multivariate analysis

**Variables entered in the final model**	**OR**	**95% CI**	**P - value**
Frequency of face washing (per day)			
>=2 times	0.3	0.1 - 1.9	NS
<2 times	1.0		
Sharing vessels during face washing			
Yes	0.4	0.03 - 4.6	NS
No	1.0		
Distance to water source (minutes)			
>20 minutes	6.5	1.8 - 16.7	0.003
<=20 minutes	1.0		
Daily household water collection (litres)			
>=60	0.4	0.1 - 0.3	<0.001
<60	1.0		
Daily household water for bathing (litres)			
>20	0.9	0.8 - 2.4	NS
<=20	1.0		
Daily household water for face washing (litres)			
>=2	0.1	0.02 - 0.7	0.02
1	1.0		
Presence of waste disposal system			
Yes	0.8	0.4- 1.6	NS
No	1.0		
Frequency of daily water collection (trips)			
>=2	0.5	0.1 - 1.8	NS
1	1.0		
Number of pre- school children in household			
yes	2.4	0.9 - 5.9	NS

## Discussion

Our data shows that in a typical Maasai village which is endemic to trachoma, a number of factors either related to individuals or households could be independently associated with active trachoma. In the present study, the increased distance to water sources and having pre-school children regardless of the number in households were significantly associated with active trachoma. Similar findings have been reported elsewhere [16]. However, not all findings agree with this observation [17]. This observation suggests that, risk factors for active trachoma vary from one epidemiological settings to another due to variations in social economic status between households, individuals or communities. In addition, variations between study designs could in part contribute to the variations of results observed in our present study and the results of the previous studies.

Other factors such as the amount of water collected in the household (> 60 litres/day) and daily amount of water allocated for face washing or bathing remained protective against active trachoma. In fact, in central Tanzania where trachoma is highly endemic, the prevalence of the disease was reported to decrease significantly in households reported to have easy access to water supply [3]. Similar observation has been reported elsewhere in Africa where trachoma is known to be endemic [17,18]. Our findings and the findings of other similar studies clearly demonstrate that, to meet the target of eliminating trachoma by 2020, targeted efforts are need to improve the availability for social needs such as water services near the trachoma endemic communities. Water availability will help to improve the household or individuals’ hygiene practices especially for face washing or bathing everyday and this in turn will help to control trachoma. Although this single approach may not be that effective but since it plays an important role on the epidemiology of the disease, its availability may significantly reduce the prevalence of the disease in endemic communities [19]. In fact in our present study, households reported to walk over a short distance (< 20 minutes) to the source of water had fewer cases of trachoma. Similar observation was reported in Tanzania [19], in which households living close to water sources had higher hygiene standard, higher water consumption and had reduced risk of acquiring trachoma [19].

Clearly, there is a strong relationship between availability of water in the endemic communities or households and the prevalence of active trachoma especially among children [12,13,19]. Our study and the findings of other previous study in Mali [10] and Tanzania [17] demonstrated that households reported to use < 2 litres of water for face washing remained susceptible to trachoma while increased the water volume and the frequency of face washing remained protective.

For the integrated approach on the control of trachoma in endemic communities, targeted public health education is required and should focus on the improvement of individual, households and entire community hygiene. For individual hygiene, bathing and face washing which reduces the contact between human and flies is the primary approach of controlling the disease especially in children [10].

On the other hand, improvement of households surrounding environment especially for deposition of animal manure, human faeces and use of closed pit latrines are important measures against the flies which act as vectors for transmitting the infection. Burning of animal dung or transferring to the farms away from the households will remove the breeding sites of the flies. Importantly, availability of health facilities close to the endemic communities will assist to relieve the morbidities and remove the reservoirs of the infections. In view of the fact that trachoma occurs in rural areas where the majority of population are highly stricken by poverty, improvement of life standard is highly required before elimination or eradication of trachoma infections can be considered a possibility in Tanzania.

Our study is subject to limitations. Some of the information used in the present study was based on self reported practices and this could have resulted into under or over estimation of the some of the findings. Sample size was calculated based on the information on water use from the pilot study. We are uncertain if the amount of water used in the household observed from the pilot study revealed the real situation in the village. If this is true may have some implications on the estimated sampling size. In addition, interpretation of the results of the present study especially the generalizability of the findings should be done with care as these findings were only observed in the small proportion of the study participants in one village.

## Conclusions

The present study has demonstrated that, distance (measured in term of time) to water sources and the presence of pre-school children regardless of the number was independently associated active trachoma at household level. Thus, improving water supply near the households and providing public health education focusing on improving individual hygiene especially in pre-school children will help to reduce the prevalence of the disease. In addition, integrating public health education with other interventions such as medical interventions remains important.

## Methods

### Study area

The present study was conducted in a Maasai village located at Hai district, north eastern Tanzania. According to the baseline trachoma survey conducted in the district, the village was reported be highly endemic to the disease and active trachoma cases are wide spread across the village [17]. The prevalence of active trachoma was high in children aged 1–9 years old (42%) [17]. At the time when the present study was conducted, the village had a total population of 800 people.

### Study design

This was a case control study conducted among households with and those without active trachoma cases. In the present study, households with two or more children aged 1–9 years with active trachoma were classified as cases while households with two or more children of similar age group without active trachoma were classified as control [13]. Active trachoma cases were defined as inflammation of the follicle tissue following the trachoma bacterial infection diagnosed clinically [20]. On the other hand, the head of the household was defined as the person who was perceived by household members to be the primary decision maker in the family, and the household was defined as individuals living together and taking meals from a common cooking facility [21].

### Sampling of study households and clinical diagnosis for trachoma

Before the survey, a list of ten cell leaders was obtained from the village administration and 10 of them were randomly selected from the list. To identify households with or without trachoma, clinical examination was carried out in all children age 1–9 years old. The inter observer agreement of the presence or absence of trachoma folliculi (TF) in children was also carried out to enhance validity of data. Before clinical examination, a list of households with or without active trachoma cases was generated with a ratio of 1:1 between cases and control. The inclusion criteria of the households were, households having five or more individuals, head of household being a Maasai, and at least two children under the age of 1–9 years old. The geographical distribution of the selected households with or without cases was also spread across the village and covered almost the entire village.

### Sample size estimation

Prior the commencement of this study, a pilot study was conducted in randomly selected households with an objective of estimating the volume of water collected and used each day by the households. The study revealed that, among the studied households, the volume of water varied between households and ranged between 60–200 litres per day.

For calculation of the sample size, the assumption was that households with active trachoma cases used 60 litres per day (30% of the maximum of the daily water use of 200 litres/day) and households without active cases of trachoma used 120 litres per day (60% of maximum of the daily water use of 200 litres/day). Assuming an (two sided) = 0.05, and power = 80% (0.80), =1-0.80 = 0.20. The sample size per group was 48 households giving total sample size of 96 households [22].

### Data collection

This study was conducted from February to August, 2005. Two trained interviewers masked against the results of clinical examination of trachoma conducted the interview using a pre-tested questionnaire. To avoid the effect of questionnaire on the response, the interviewers ensured consistency on asking the questions. To answer the objectives of the study, the questionnaires were only administered to household mothers or women who were allowed by the household heads to participate in the study. In the present study setting, only household mothers or women of child bearing age participated in all domestic activities within the household. The questionnaire had questions regarding the general household or individual hygiene practices including presence and use of pit latrines, use of soap for bathing (if children used soap during bathing or washing clothes) and washing, facial cleanliness (face and eyes), quantity of water collected and used for different purposes at home (litres/day). Also, the questionnaire explored information on the use of clean towel (towel used to dry the face after washing), sharing of towel or vessels among children, frequency of face washing (self- reported) and frequency of bathing children (by reporting the number of times a day). Information on distance to water sources (measured in term of minutes), the type of water sources (well, rivers and other sources) were also collected. In addition, information on the means used to transport collected water from the source to the households was collected. The amount of water collected each day by the households was reported by the respondents; however, the trained interviewers estimated the quantity in term of litres per day based on the volume of the containers used to collect water from the sources.

### Data analysis

Data were analyzed using the Statistical Package for the Social Sciences (SPSS) version 17.0 for windows (SPSS Inc., Chicago, IL, USA). Cross tabulations were used to detect associations and to select the most relevant variables for inclusion in a logistic regression model. Univariate analysis was done to calculate odds ratio (OR) and their 95% confidence interval and chi-square (and chi-square for trend) were also calculated. Since some of the water variables (such as amount of water collected, frequency of water collection) were likely to be inter-related, a multivariate analysis was conducted to estimate the strength of the associations while controlling for a number of confounding variables.

A multivariate logistic model was used to identify the independent association of hygiene risk factors and active trachoma. Three different models were carried out as a number of parameters were likely to be strongly associated with each other. In the first model, all variables associated (*P*<0.05) with active trachoma using univariate analysis were included while in the second model only variables that remained statistically significant in the first model were included. In the third (final) model all parameters in the first model were analyzed using a forward stepwise logistic regression so that the relative contribution of each parameter could be assessed separately and in sequence.

### Ethical considerations

Ethical approval was obtained from the Ethical Committee of Kilimanjaro Christian Medical College of Tumaini University, Tanzania. The committee allowed the use of both verbal and written informed assents and consents. Informed written or oral consents were obtained from all the household mothers or women prior to inclusion into the study. For the > 10 years old children, the parents/guardians/caregivers gave written informed consent for them to participate in the study.

## Competing interests

All authors declare they have no competing interest in this study.

## Authors’ contributions

MJM conceived, designed, implemented the study and supervised data entry. MJM, EJK and HDM did data analysis and interpretation. MJM and EJK wrote the manuscript. EJK, MJM and HDM revised the manuscript critically. All authors approved the final version for submission.

## Ethical clearance

This study was granted ethical approval by Kilimanjaro Christian Medical College University research ethics committee. Hai district health office approved the study to be conducted in the Roselini village in Hai district. All participants were given written consent before participated in the study

## Supplementary Material

Additional file 1 Multilingual abstracts in the six official working languages of the United Nations. Click here for file
