# Supplementary material for: Association between water related factors and active trachoma in Hai district, Northern Tanzania
Source: Infect Dis Poverty. 2012 Nov 1;1:10. doi: 10.1186/2049-9957-1-10 (PMC3710161; doi:10.1186/2049-9957-1-10)
Supplement: Additional file 1 — Multilingual abstracts in the six official working languages of the United Nations. [file 2049-9957-1-10-S1.pdf]

Translation of the abstract into the six official working languages of the United Nations

الارتباط بين العوامل المتعلقة بالمياه والتراخوما النشطة في منطقة هاي في شمال تنزانيا

مايكل ج. ماهاند، همفري د. مازيغو، إلننغايا ج. كويكا

الملخص

الخلفية: تنتشر التراخوما على نطاق واسع جنوب الصحراء الكبرى في أفريقيا، وهي ترتبط بشكل رئيسي مع عدم التمكن من الوصول إلى مصادر المياه. ومع ذلك، فإن هذا الارتباط لم يمكن إظهاره على الاطلاق في بعض المجتمعات، وخاصة في شمال تنزانيا. و لسدّ هذه الثغرة، تم إجراء دراسة الحالات والشواهد الحالية لتقييم الارتباط بين العوامل ذات الصلة بالمياه والنظافة العامة وبين التراخوما النشطة لدى الأطفال في سن ما قبل المدرسة وسن المدرسة في حي هاي في شمال تنزانيا.

النتائج: كانت الأسر التي ذكرت أنها تستخدم> 60 لترا من المياه يوميا أقل عرضة للإصابة بالمرض النشط (نسبة الأرجحية = 0.4 ، فاصلة الثقة 95%: 0,1 – 0,3؛ الاحتمالية <0.001) مقارنة بالأسر التي تجمع ≤ 60 لترا. تزداد إمكانية الإصابة بالتراخوما مع ازدياد البعد عن مكان وجود الماء (نسبة الأرجحية = 6,5، فاصلة الثقة 95%: 1,8 – 16,7 ؛ الاحتمالية = 0.003). أفراد الأسرة الذين ذكروا أنهم يستخدمون <2 لتر من الماء لغسل الوجه أكثر عرضة للإصابة بالتراخوما (نسبة الأرجحية = 5,12، ، فاصلة الثقة 95% : 1,87 – 14,6، الاحتمالية = 0,001). كما ارتبط ازدياد عدد الأطفال قبل سن المدرسة الموجودين في المنزل مع زيادة إمكانية الإصابة بالتراخوما النشطة بمقدار 2،46 ضعفا.

الاستنتاجات: تحسين إمدادات المياه بالقرب من أ الأسر، وتوفير التوعية الصحية العامة مع التركيز على تحسين الوضع الاجتماعي والاقتصادي والنظافة الفردية لأفراد الأسر وخاصة بين الأطفال قبل سن المدرسة سيساعد جزئيا على إنقاص معدل انتشار المرض.كما أن دمج برامج التوعية الصحية العامة مع التدخلات الأخرى مثل التدخلات الطبية تبقى أمرا هاما.

Translated from English version into Arabic by Lina SM, through

TRANSLATORS WITHOUT BORDERS

坦桑尼亚北部 Hai 地区活动期沙眼与水相关因素的关系

Michael J. Mahande, Humphrey D. Mazigo, Eliningaya J. Kweka

摘要

背景：沙眼在撒哈拉以南的非洲地区广泛分布，主要与水资源稀缺有关。然而，这一相关性从未在坦桑尼亚北部的人群中加以论证。为弥补该空缺，在坦桑尼亚北部 Hai 地区对学龄前儿童和学龄儿童开展病例对照研究，评价水相关因素、整体卫生情况和活动期沙眼的相关性。

结果：家庭自报每日用水> 60 升的儿童较≤ 60 升家庭的儿童更不易患活动期沙眼 (OR= 0.4, 95% CI: 0.1 - 0.3; P<0.001)。患病风险随离取水点的距离的增加而上升 (OR= 6.5, 95% CI; 1.8 - 16.7; P= 0.003)。家庭成员中有用< 2 升水洗脸的儿童更容易患病(OR= 5.12, 95% CI: 1.87-14.6, P = 0.001)。家庭中学龄前儿童越多，儿童患活动期沙眼的风险增加，约 2.46 倍。

结论：改善居民家庭附近的水源供应，针对改善家庭社会经济状态和个人卫生，尤其是学龄前儿童的卫生状况，开展公共卫生教育将在一定程度上有助于减少沙眼的流行。此外，将其他干预措施，例如药物干预与公共卫生教育相结合仍然非常重要。

Translated from English version into Chinese by Qu Lin-Ping, through

CDC CHINESE CENTER FOR DISEASE CONTROL AND PREVENTION

NATIONAL INSTITUTE FOR PARASITIC DISEASES

Trachome actif associé aux facteurs liés à l'eau dans le district de Hai dans le nord de la Tanzanie

Michael J. Mahande, Humphrey D. Mazigo, Eliningaya J. Kweka

Résumé

Contexte : le trachome, largement répandu en Afrique subsaharienne, est principalement lié à un accès insuffisant à l'eau. Cependant cette association n'a jamais été démontrée dans certaines communautés, principalement dans le nord de la Tanzanie. Pour pallier ce manque, la présente étude cas-témoins a été menée pour évaluer le lien entre les facteurs liés à l'eau, l'hygiène générale et le trachome actif chez les enfants en âge préscolaire et scolaire dans le district de Hai, situé dans le nord de la Tanzanie.

Résultats : Les familles qui ont déclaré avoir une consommation d'eau supérieure à 60 litres par jour étaient moins susceptibles de contracter la maladie active (OR = 0,4, IC 95 % ; 1,1 - 0,3 ; P < 0,001) que les foyers ayant une consommation inférieure ou égale à 60 litres. Le risque de contracter un trachome augmente avec la distance avec l'éloignement du point d'eau (OR = 6,5, IC 95 % ; 1.8 - 16.7 ; P = 0.003). Les membres des foyers qui ont déclaré utiliser moins de 2 litres d'eau pour se laver le visage étaient davantage susceptibles d'être trachomateux (OR = 5.12, IC 95 % : 1.87 - 14.6, P = 0.001). L'augmentation du nombre d'enfants en âge préscolaire dans les foyers a aussi été associée avec un risque de trachome actif multiplié par 2,46.

Conclusion : l'amélioration de l'accès à l'eau près des foyers et la promotion de l'éducation à la santé publique en mettant l'accent sur l'amélioration du statut socio-économique des foyers et l'hygiène individuelle, en particulier chez les enfants en âge préscolaire, pourra aider en partie à réduire la prévalence de cette maladie. Il est important d'ajouter à cela l'intégration de l'éducation à la santé publique ainsi que d'autres interventions, médicales par exemple.

Translated from English version into French by C. Santamaria, through

TRANSLATORS
WITHOUT BORDERS

Взаимосвязь зависящих от воды факторов и активной формой трахомы в районе Хай северной Танзании

Майкл Махандэ, Хамфрэй Мазиго, Элинингайа Квека

Краткое описание

Справочная информация: Трахома широко распространена в странах Африки, расположенных южнее Сахары, причиной которой, в основном, является скудная доступность воды. Однако данная связь никогда не оглашалась в некоторых общинах, в особенности в северной Танзании. Для того, чтобы заполнить этот пробел, в районе Хай северной Танзании среди детей дошкольного и школьного возраста методом "случай - контроль" было проведено данное исследование, целью которого было определить взаимосвязь зависящих от воды факторов, общей гигиены и активной формой трахомы.

Результаты: В семьях, использующих >60 литров воды в день, активная форма болезни менее вероятна (OR=0,4, 95% CI: 0,1 - 0,3; P<0,001), в отличие от семей, расходующих ≤60 литров. Риск заражения трахомой возрастает с увеличением расстояния от места доступа к воде (OR=6,5, 95% CI; 1,8 - 16,7; P=0,003). Члены семей, сообщившие о том, что используют <2 литров воды для умывания, вероятнее всего являются носителями трахомы (OR=5,12, 95% CI: 1.87-14.6, P = 0.001). Увеличение числа детей дошкольного возраста в домовладении также имеет отношение к увеличивающемуся риску заболевания в 2,46 раза.

Выводы: Улучшение водоснабжения вблизи жилых домов и обучение здравоохранению с особым упором на аспекты улучшения социально-экономического статуса семьи и личной гигиены, в особенности детей дошкольного возраста, будут способствовать снижению распространения заболевания. Кроме того, внедрение санитарного просвещения населения с другими мероприятиями, такими как медицинское вмешательство, остается очень важным.

Translated from English version into Russian by Yulia Novikova-Wythe, through

TRANSLATORS
WITHOUT BORDERS

Asociación entre el factores relacionados con el agua y el tracoma activo en el distrito de Hai, norte de Tanzania

Michael J. Mahande, Humphrey D. Mazigo, Eliningaya J. Kweka

Resumen

Antecedentes: El tracoma es una enfermedad muy extendida en el África Subsahariana y se asocia principalmente con el escaso acceso al agua. Sin embargo, esta asociación nunca ha sido demostrada en algunas de las comunidades, especialmente en el norte de Tanzania. Para cubrir esta brecha, se realizó este estudio de control de casos para determinar la asociación de factores relacionados con el agua, la higiene general y el tracoma activo en niños de edad preescolar y escolar del distrito de Hai, en el norte de Tanzania.

Resultados: Se halló que las familias que informaron el uso de > 60 litros de agua por día fueron menos propensas a desarrollar la enfermedad (OR= 0,4; 95% CI: 0.1 – 0,3; P<0,001) en comparación con los grupos familiares que informaron el uso de ≤ 60 litros. El riesgo de tracoma aumentó con el aumento en la distancia al punto de recolección de agua (OR= 6,5; 95% CI; 1,8 – 16,7; P= 0,003). Los miembros de los grupos familiares que informaron el uso de < 2 litros de agua para el lavado de la cara fueron más propensos al tracoma (OR= 5,12; 95% CI: 1,87-14,6, P = 0,001). También se asoció un incremento en el número de niños en edad preescolar en el hogar con un incremento de 2,46 veces en el riesgo de tracoma activo.

Conclusiones: Mejorar el suministro de agua cerca de los hogares y proporcionar educación en salud pública que se enfoque en las mejoras de las condiciones socio-económicas de los grupos familiares y la higiene individual, especialmente en los niños de edad preescolar, en parte ayudará a reducir la prevalencia de la enfermedad. Adicionalmente, integrar la educación en salud pública con otras intervenciones, como intervenciones médicas, continúa siendo importante.

Translated from English version into Spanish by Aldana Gómez Ríos, through

TRANSLATORS
WITHOUT BORDERS
